# The incidence of meniscal cyst formation following meniscal repair using the all‐inside suture anchor device is comparable to conventional techniques

**DOI:** 10.1002/jeo2.70049

**Published:** 2024-10-08

**Authors:** Kazumi Goto, Takaki Sanada, Eisaburo Honda, Shin Sameshima, Miyu Inagawa, Yutaro Ishida, Koji Matsuo, Ryota Kuzuhara, Hiroshi Iwaso

**Affiliations:** ^1^ Department of Sports Orthopedic Surgery Kanto Rosai Hospital Kanagawa Japan

**Keywords:** all‐inside suture, anchor device, knee, meniscal cyst, meniscal repair

## Abstract

**Purpose:**

Post‐operative meniscal cyst formation occurs following all‐inside device meniscal repair. This study aimed to compare the incidence of cysts in patients who underwent meniscal repair with and without all‐inside suture devices.

**Methods:**

This retrospective study included 227 knees that underwent meniscal repair between 2021 and 2022. The incidence of post‐operative meniscal cysts was compared between patients who underwent repair using an all‐inside suture anchor device (Group SA) and those who did not use an anchor (Group NA), based on post‐operative magnetic resonance imaging (MRI) findings. Risk factors, such as the number of anchors used, were investigated. Using a subgroup analysis, the incidence of meniscal cysts based on the type of device used was investigated.

**Results:**

Groups SA and NA comprised 125 and 102 knees, respectively. Group SA had 11 cases of cysts (9% incidence), whereas Group NA had 7 cases (7% incidence), and no statistically significant difference was observed (*p* = 0.63). Symptomatic cysts were observed in two patients (1.6%) in Group SA, whereas none was observed in Group NA (0%); the difference was not significant (*p* = 0.50). Factors such as the number of anchors and sutures used and MRI timing were not identified as risk factors. Cyst incidence varied according to anchor type: Stryker AIR+ (4 out of 55, 7%), Smith & Nephew Fast‐Fix 360 (7 out of 56, 13%) and Arthrex Fiber Stitch (0 out of 26, 0%), with no significant difference found (*p* = 0.14).

**Conclusion:**

The incidence of cysts in patients undergoing meniscal repair with an all‐inside suture anchor device was 9%, showing no significant difference compared with Group NA. Cyst incidence was not affected by device type.

**Level of Evidence:**

Level III, retrospective comparative study.

**Abbreviations**
ACL: anterior cruciate ligament
I‐O: inside‐out
LM: lateral meniscus
MM: medial meniscus
MRI: magnetic resonance imaging
O‐I: outside‐inside
STIR: Short Tau Inversion Recovery

## INTRODUCTION

The all‐inside suture anchor device for meniscal repair offers the advantages of simplicity and high reproducibility, with clinical outcomes comparable to conventional methods, such as the inside‐out (I‐O) technique [6, 7, 10, 15, 20]. However, complications, such as iatrogenic nerve injury and intra‐articular anchor displacement, have been reported [8, 9, 10, 14]. Recently, post‐operative meniscal cyst formation was reported to occur at a higher rate with all‐inside devices (29%–40%) than with the I‐O technique (1.7%–8.3%) [21, 24]. Generally, most meniscal cysts are asymptomatic and often discovered incidentally on imaging [10]. However, they can cause knee pain during physical activity, and serious complications such as peroneal nerve palsy associated with meniscal cysts and erosion into the tibial plateau have also been reported [3, 4, 12]. Therefore, these complications should be avoided whenever possible.

In recent years, newer generation devices, including all‐inside devices utilising soft‐type anchors called all‐suture anchors, or anchors and needles with shapes smaller than conventional ones, have emerged [1]. Previous reports on meniscal cysts have investigated only two products, the Ultra Fast‐Fix (Smith & Nephew) and Fast‐Fix 360 (Smith & Nephew) [21, 24], leaving the occurrence of meniscal cysts with other all‐inside suture device products unknown. The primary objective of this study is to determine the incidence of meniscal cysts following meniscal repair using all‐inside devices, including newer‐generation devices, and to compare this incidence with that observed in repairs without the use of anchor devices. Additionally, the secondary objectives of this study are to investigate the impact of different types of all‐inside devices on the incidence of meniscal cysts and to identify potential risk factors associated with cyst formation, such as the number of anchors used and the timing of post‐operative magnetic resonance imaging (MRI). This study hypothesises that the incidence of cysts in meniscal repair using all‐inside anchor devices will be comparable to that observed in repairs without anchor devices, particularly when using newer‐generation devices. The clinical relevance of this study lies in understanding the cyst incidence and associated risks with all‐inside suture anchor devices, providing insight into the appropriate suture method for meniscal injuries and offering useful information when selecting all‐inside devices.

## MATERIALS AND METHODS

### Patient cohorts

**Figure 1:**
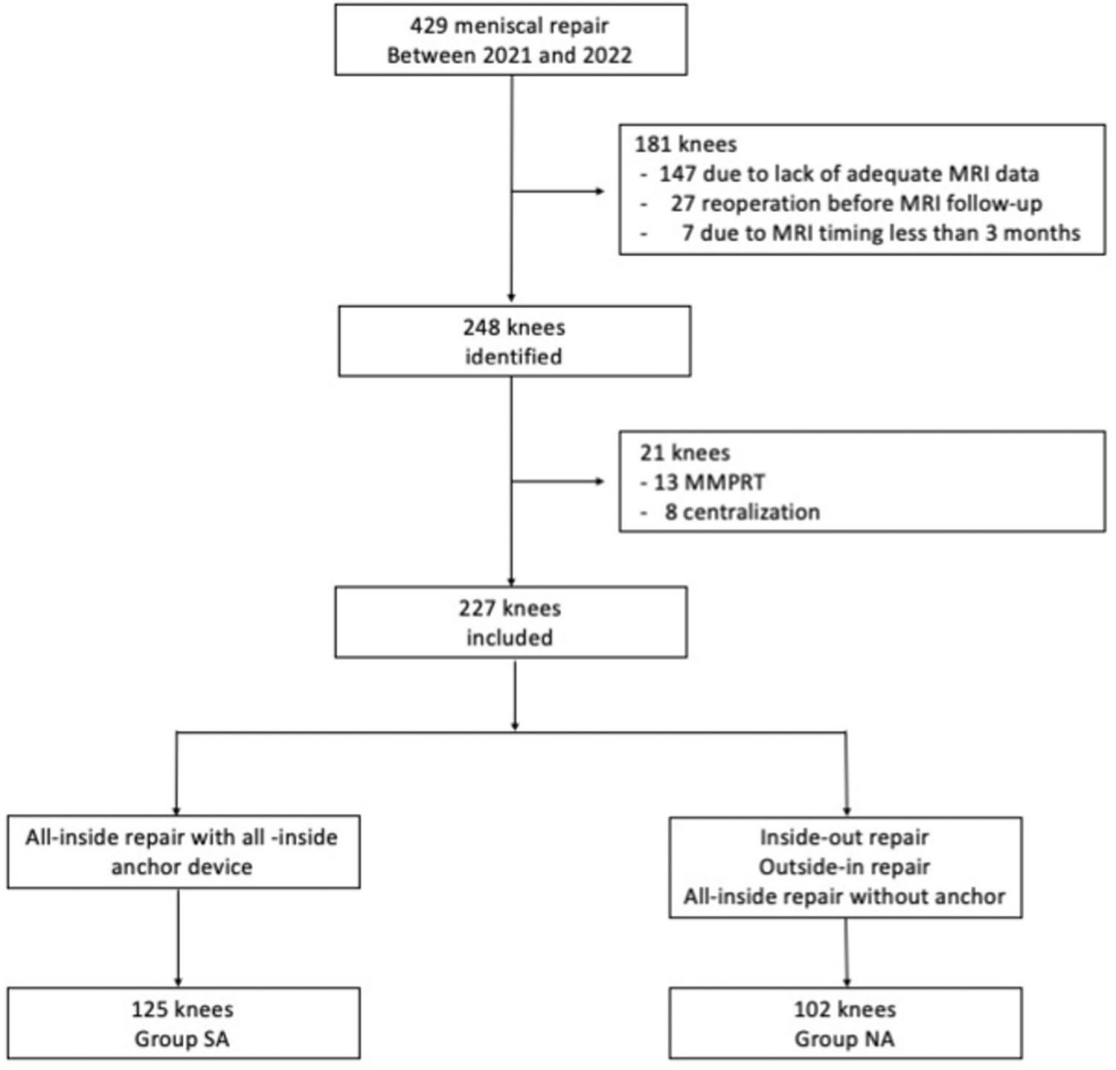
Flowchart illustrating the criteria for exclusion from the study. MMPRT, medial meniscus posterior root tear; MRI, magnetic resonance image.

Our institutional review board approved this retrospective comparative study. In total, 429 knees that underwent meniscal repair at our institution between January 2021 and December 2022 were enrolled in this study. Knees that underwent MRI at least 3 months post‐operatively were included. Patients with preoperative meniscal cysts on MRI, a history of arthroscopy, multiple ligament injuries, repair of the medial meniscus (MM) posterior root tear or centralisation repair were excluded [17]. In addition, patients with sutured meniscus re‐rupture or those requiring reoperation of the meniscus before follow‐up MRI were excluded. In total, 227 knees were included in this study (Figure 1).

Knees were separated into two groups: Group SA, comprising the knees that underwent meniscal repair using the all‐inside suture anchor device, and Group NA, comprising those that underwent repair without using the all‐inside suture anchor. This study investigated and compared the incidence of cysts and symptomatic cysts between both groups. In addition, as part of the exploratory research, a multivariate analysis was conducted to determine whether age, sex, side (left/right), location (lateral meniscus [LM] or MM, anterior horn/body/posterior horn), type of tear (trauma injury or degenerative tear, longitudinal/radial/horizontal/complex), presence of a discoid meniscus, sports level (Tegner Activity Scale), number of anchors used, number of sutures and follow‐up timing of MRI examination were risk factors. Furthermore, differences in meniscal cyst incidence based on the type of device used were investigated as a sub‐analysis.

The primary outcome of this study was the incidence of meniscal cysts following meniscal repair using all‐inside suture anchor devices, compared between Group SA and Group NA. Secondary outcomes included the incidence of symptomatic cysts between the two groups and an analysis of potential risk factors for meniscal cyst formation. Additionally, a sub‐analysis was conducted to compare the incidence of meniscal cysts based on the type of all‐inside suture anchor device used, including the Fast‐Fix 360, Stryker AIR+ (Stryker) and Arthrex Fiber Stitch (Arthrex).

### Surgical technique

Surgery was performed by multiple surgeons. The decision to perform meniscectomy or repair is made individually, considering the patient's background, preferences and type of tear. Generally, for longitudinal tears of the MM in the red zone measuring ≥1.5 cm and exhibiting significant instability, the repair was performed using the I‐O technique. In cases where the approach was challenging, such as anterior horn tears, the outside‐inside (O‐I) technique was utilised. Tears <1.5 cm long or those longer than 1.5 cm but without significant instability were managed intraoperatively based on a comprehensive assessment, typically using the all‐inside suture anchor device. In addition, all‐inside devices were employed for posterior segment tears, where the I‐O technique poses challenges. The choice of the all‐inside suture anchor device was based on the surgeon's preference and included options such as Fast‐Fix 360, Styler AIR+ and Arthrex Fiber Stitch. For LM tears, attempts were made to achieve true all‐inside repair using a 2‐0 Fiber Wire (Arthrex) and an antegrade suture passer such as the Knee Scorpion (Arthrex) whenever possible, as documented in previous studies [5, 19]. However, when adequate working space was not obtained, or for tears in which suturing with the true all‐inside technique was challenging, approaches such as the all‐inside device, I‐O technique or O‐I technique were selected, depending on the case.

### Post‐operative rehabilitation

Based on the type of tear, they were classified into four categories: MM stable/unstable and LM stable/unstable. Rehabilitation was carried out according to the protocol outlined in Table 1. In cases of discoid LM, the LM protocol was delayed by 2 weeks. However, the protocol serves as a guideline. Based on the stability confirmed during surgery and at the surgeon's discretion, the rehabilitation schedule can be flexibly adjusted according to the patient's background.

**Table 1:** Post‐operative rehabilitation protocol.

		MM		LM	
		Stable	Unstable	Stable	Unstable
ROM	0–4 W	0–120°	0–120°	0–120°	0–90°
	4–8 W	Full	Full	0–120°	0–120°
	8 W+			Full	Full
Weight‐bearing	0–2 W	Full	Toe touch	Full	Toe touch
	2–4 W		1/2PWB		1/2PWB
	4 W+		FWB		FWB
Exercise bike		3 W	6 W	1 M	6 W
Stair workout		1.5 M	2 M	1.5 M	2 M
Leg extension		1 M	1.5 M	1 M	1.5 M
Jogging		2 M	3 M	2 M	3–4 M
Step exercise		2.5 M	3.5 M	2.5 M	3.5 M
RTS		3 M	4 M	3 M	4–5 M

*Note*: A stable tear was defined as a longitudinal tear in the red zone or red‐white zone, whereas an unstable tear was defined as a longitudinal tear in the white zone, a horizontal tear in the red‐white zone or white zone, or a radial tear.

Abbreviations: FWB, full weight‐bearing; LM, lateral meniscus; M, months(s); MM, medial meniscus; PWB, partial weight‐bearing; ROM, range of motion; RTS, return‐to‐sports; W, week(s).

### Radiological evaluation

**Figure 2:**
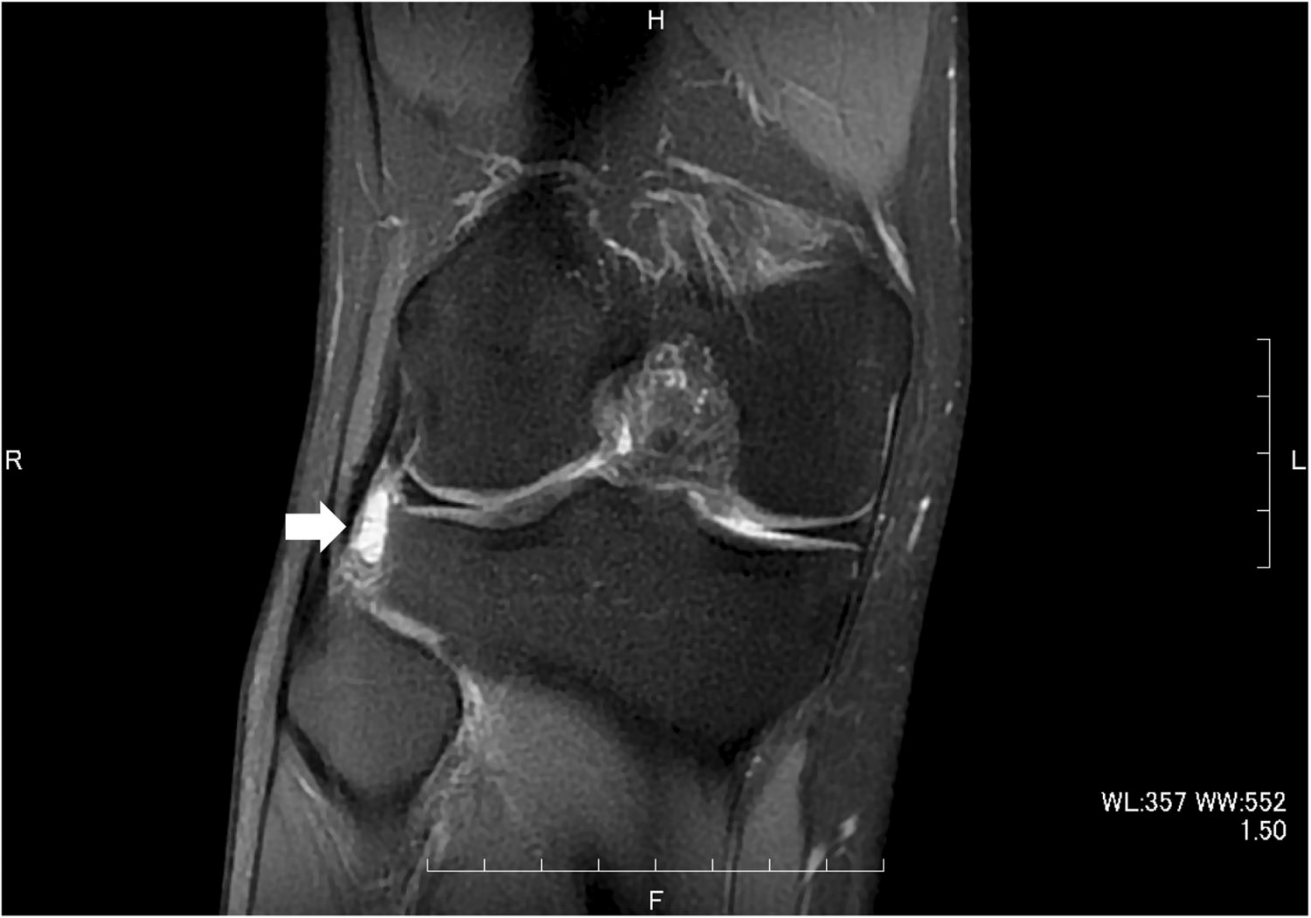
The finding of a post‐operative meniscal cyst (white arrow) in the coronal STIR image of the MRI. MRI, magnetic resonance imaging; STIR, Short Tau Inversion Recovery.

At the institution where the study was conducted, MRI examinations were performed using a 3.0‐T MRI system (MAGNETOM Skyra, Siemens) or a 1.5‐T MRI system (SIGNA Artist, GE). An MRI was deemed appropriate for the study if it met the following conditions: both the coronal and sagittal planes were created in a 1.5‐ or 3.0‐T MRI, and a Short Tau Inversion Recovery (STIR) image was taken. MRI scans performed at other institutions that met the criteria were included in the analyses. A cyst was defined as fluid accumulation around the anchor in the STIR image with a high signal of at least 5 mm visible in both the coronal and sagittal views (Figure 2) [21]. An orthopaedic surgeon conducted all analyses. A total of 20 knees were randomly selected to verify the accuracy regardless of the presence of cysts, and consensus on cyst presence was examined by three individuals, resulting in a 100% agreement rate.

### Clinical evaluation

Medical records were retrospectively examined to determine whether the patients' symptoms were associated with cysts. If it was unclear whether the cysts caused the symptoms, the patients were not identified as having symptomatic cysts.

### Statistical analysis

All statistical analyses were performed using the R software (version 4.2.1; R Development Core Team). Continuous variables are reported as means and standard deviations, whereas categorical variables are presented as percentages. The Shapiro–Wilk test of normality was used to determine whether the data were normally distributed. The rate of post‐operative meniscal cyst formation between the groups was compared using the Pearson chi‐square test. Logistic regression analysis was employed to identify risk factors associated with meniscal cysts. Subgroup analysis involved using Fisher's exact test to determine the incidence rates of cysts and symptomatic cysts among the three types of anchors. Differences in patient backgrounds among the three groups in the subgroup analysis were assessed using either one‐way analysis of variance or the Kruskal–Wallis test, depending on the normal distribution of continuous variables. Statistical significance was set at *p* < 0.05. A minimum sample size of 21 patients in each cohort was required to provide appropriate power (*β* = 0.80) at a significance level of 0.05. This calculation accounted for the difference in the incidence of post‐operative meniscal cyst formation between I‐O and all‐inside device techniques, as reported in a previous study [21].

## RESULTS

Demographic data are presented in Table 2. Group SA and Group NA comprised 125 and 102 knees, respectively. The incidence of cysts was 11 cases (9%) in Group SA and 7 cases (7%) in Group NA, with no statistically significant difference observed (*p* = 0.63). In Group SA, symptomatic cysts were observed in two cases (1.6%), whereas Group NA had 0 cases (0%). No significant difference was detected (*p* = 0.50). Sex, age, laterality, site (MM or LM), number of anchors and sutures used and MRI timing were not identified as apparent risk factors (Table 3). The breakdown and results of the subgroup analyses are presented in Table 4. Cyst incidence according to anchor type was 4 out of 55 (7%) with AIR+, 7 out of 56 (13%) with Fast‐Fix 360 and 0 out of 26 (0%) with Fiber Stitch, with no significant difference observed (*p* = 0.14). No differences were observed in patient backgrounds among these subgroups, except for the MRI timing (Table 4).

**Table 2:** Patient demographics.

	Group SA (*n* = 125)	Group NA (*n* = 102)	*p*
Age (years)	28.9 ± 14.5	25.6 ± 13.6	0.079
Sex (female/male)	47/78	35/67	0.677
Side (left/right)	71/54	53/51	0.425
Height (cm)	167.1 ± 10.0	165.0 ± 10.6	0.136
Body weight (kg)	67.5 ± 21.0	60.4 ± 13.8	0.004
Tegner activity scale	6 [4–7]	7 [4–8]	0.244
ACL injury	14 (11.2%)	7 (6.9%)	0.358
MM/LM/DLM	50/50/23	35/36/34	0.064
Trauma injury/degenerative tear	117/8	98/4	0.554
Location (anterior horn/body/posterior horn)	29/89/98	45/92/45	<0.01
Type (longitudinal/radial/horizontal/complex)	124/22/19/17	93/36/18/2	<0.01
Timing of MRI after surgery (days)	299 [198–368]	268 [169–360]	0.079

Abbreviations: ACL, anterior cruciate ligament; DLM, discoid lateral meniscus; LM, lateral meniscus; MM, medial meniscus; MRI, magnetic resonance imaging.

**Table 3:** Results of the logistic regression analysis.

Parameter	Odds ratio (95% CI)	*p*
Age (year)	1.15 (0.95–1.38)	0.142
Male	2.7 (0.079–92.20)	0.581
Right side	104 (0.22–49,000)	0.140
MM	120 (0.09–157,000)	0.191
Number of AI devices	1.84 (0.66–5.17)	0.246
Number of sutures	0.53 (0.21–1.33)	0.175
Timing of MRI (days)	1.01 (0.92–1.02)	0.349

Abbreviations: AI, all‐inside; CI, confidence interval; M, medial meniscus; MRI, magnetic resonance imaging.

**Table 4:** Incidence of meniscal cyst formation resulting from the all‐inside suture anchor device.

	Fast‐Fix 360 Smith & Nephew	AIR+ Stryker	Fiber Stitch Arthrex	*p*
Total number	56	55	26	–
Cyst	7	4	0	0.139
Symptomatic Cyst	2	0	0	0.669
Age	28.9 ± 16.1	30.8 ± 14.3	27.3 ± 14.8	0.525
Sex	22/34	16/39	14/12	0.105
Side (L/R)	35/21	32/23	12/14	0.374
Timing of MRI (days)	237 [184–348]	357 [215–383]	356 [228–372]	0.021

Abbreviations: L/R, left or right; MRI, magnetic resonance imaging.

## DISCUSSION

The most important finding of this study is that no difference was observed in post‐operative meniscal cyst formation between the all‐inside device and the method without anchors. Specifically, the incidence of cyst formation was 9% in the all‐inside group compared to 7% in the non‐anchor group, indicating that newer‐generation all‐inside devices may not increase the risk of cyst formation compared to traditional suture methods. This result is clinically significant, as it suggests that the adoption of all‐inside techniques for meniscal repair does not compromise patient safety with respect to cyst formation. Furthermore, factors such as the timing of MRI scans and the number of sutures used were not identified as significant risk factors for cyst development. These findings contribute to the growing body of evidence supporting the safety profile of all‐inside meniscal repair methods.

The all‐inside‐device technique for meniscal suturing has become the mainstream approach in meniscal repair because of its advantages, such as shorter surgical time, reduced need for additional incisions and a lower risk of nerve damage compared to the I‐O technique [2, 7, 8, 9, 14, 18]. Biomechanical studies have demonstrated that all‐inside devices provide suture strength and clinical outcomes comparable to the I‐O technique [1, 6, 10, 19]. Despite these benefits, drawbacks include a higher failure rate after 5 years post‐operatively compared to the I‐O technique, a higher rate of implant‐related complications and increased costs resulting from device malfunction and the need for multiple anchors [5, 10, 22, 23]. In addition to these complications, recent studies have highlighted a higher incidence of meniscal cysts following meniscal repair using all‐inside device anchors. One study reported that 40% of menisci (14 out of 30) sutured with the all‐inside technique developed cysts, compared to only 1.7% (1 out of 60) with the I‐O technique, with symptomatic cysts occurring in 6.7% of all‐inside cases [21]. Similarly, Terai et al. found a 29% cyst incidence (15 out of 51) with symptomatic cases in 6.5% using the Fast‐Fix system [24]. These previous studies have primarily focused on older‐generation all‐inside devices, such as the Fast‐Fix and Ultra Fast‐Fix, which are associated with higher rates of meniscal cyst formation because of their larger profiles and increased rigidity.

In contrast, newer‐generation devices like the Fast‐Fix 360, AIR+ and Fiber Stitch feature lower‐profile implants, finer needles, and different anchor types, which may reduce mechanical irritation and subsequent inflammatory responses [1, 2, 16, 19]. Kinoshita et al. reported a significantly lower cyst occurrence rate with these newer devices (5.56%) compared to older models (26.4%) [16]. In the current study, the incidence of meniscal cysts with the all‐inside technique was 9% (11 out of 125), with symptomatic cysts in 2.4% (3 out of 125). These results support the hypothesis that meniscal cyst formation is more likely with older, bulkier anchors, potentially because of synovial fluid leakage through small communication holes created during the suturing process or the body's inflammatory response to foreign materials [3, 4, 11, 24]. The fact that no significant difference in cyst incidence was observed between all‐inside and no‐anchor techniques in this study suggests that newer, low‐profile all‐suture anchors might effectively minimise these risks. This underscores the importance of continuous innovation in device design to improve patient outcomes and reduce post‐operative complications.

This study had some limitations. First, it is a retrospective study. Device selection by multiple surgeons depends on the tear morphology and location and will likely influence the outcomes. In addition, significant differences in the location and type of meniscal injuries between the two groups may also have affected the results. Second, this study included patients with concomitant anterior cruciate ligament (ACL) injuries. Cases in which ACL reconstruction surgery was performed simultaneously have a high healing rate, potentially affecting the cyst formation rate [13]. However, considering that there was no difference in the concomitant ACL injury rates between the two groups (Table 2), the impact was considered minimal. Third, the definition of cyst is ambiguous, and the MRI protocols are not strictly standardised. Previous studies have not quantitatively defined or validated cysts. Nishino et al. defined cysts as 5 mm or larger in both planes [21], but smaller cysts with symptoms have been reported in past studies [3, 4], and limiting the definition to only larger ones might exclude clinically relevant cysts. Fourth, this study combined I‐O, O‐I and true all‐inside techniques, each with entirely different suturing methods, into Group NA. A true all‐inside technique does not create holes in the joint capsule; therefore, the rate of cyst formation may be low. However, no significant difference in the post‐operative cyst formation rate was observed between the device and non‐device groups. Finally, the timing of the MRI scans varied greatly, which may have influenced the results. Terai et al. noted that 80% of cysts were detected within 1 year [24], suggesting that MRI timing had minimal influence on the outcomes. In the current study, MRI timing was not identified as a significant risk factor.

Despite these limitations, this study is the first to investigate the cyst formation rate after meniscal repair, including newer‐generation anchors, and to compare it with suturing methods without all‐inside anchors, encompassing more cases than previous studies. These findings show that using all‐inside devices does not change the occurrence rate of meniscal cysts and that the occurrence rate does not vary depending on the type of device selected from the new generation of anchors, providing clinically useful information.

## CONCLUSION

The incidence of cysts in patients undergoing meniscal repair with an all‐inside suture anchor device was 9%, whereas that in the group without an anchor was 7%, with no significant difference. MRI timing and the number of devices were not identified as risk factors. There were no differences in the cyst incidence rates among the three devices used. Clinically, the study demonstrated that the all‐inside anchor device did not significantly increase the post‐operative cyst formation rate and showed safety equivalent to that of conventional methods, such as the I‐O technique. Furthermore, no difference was observed in the cyst incidence rates among all the investigated devices, suggesting that the risk of meniscal cysts may not increase with the use of any all‐inside device according to the surgeon's preference.

## AUTHOR CONTRIBUTIONS

Kazumi Goto conceived the presented idea. Hiroshi Iwaso and Takaki Sanada developed the theory and performed the computations. Kazumi Goto and Takaki Sanada verified the analytical methods. Hiroshi Iwaso encouraged Kazumi Goto to investigate this study and supervised the findings of this work. Kazumi Goto designed the study and wrote the initial draft of the manuscript. Takaki Sanada contributed to the analysis and interpretation of data and assisted in the preparation of the manuscript. All other authors have contributed to data collection and interpretation, and critically reviewed the manuscript. Hiroshi Iwaso conceived of the study and oversaw the overall direction and planning. All authors approved the final version of the manuscript and agree to be accountable for all aspects of the work in ensuring that questions related to the accuracy or integrity of any part of the work are appropriately investigated and resolved.

## CONFLICT OF INTEREST STATEMENT

The authors declare no conflict of interest.

## ETHICS STATEMENT

This study was approved by our institutional review board. Written informed consent was obtained from the patients for participation in this case series.

## Data Availability

The data sets during and/or analysed during the current study are available from the corresponding author upon reasonable request.
